# Polymorphisms in Fc Gamma Receptors and Susceptibility to Malaria in an Endemic Population

**DOI:** 10.3389/fimmu.2020.561142

**Published:** 2020-11-12

**Authors:** Mireille Ahou Amiah, Amed Ouattara, David Tea Okou, Simon-Pierre Assanvo N’Guetta, William Yavo

**Affiliations:** ^1^ Malaria Research and Control Center, National Public Health Institute, Abidjan, Côte d’Ivoire; ^2^ Laboratory of Genetics, Unité de Formation et de Recherche (UFR) BIOSCIENCES, Félix Houphouët-Boigny University, Abidjan, Côte d’Ivoire; ^3^ Malaria Research and Training Center, University of Sciences, Techniques and Technologies, Bamako, Mali; ^4^ Department of Pediatrics, Emory University School of Medicine, Atlanta, GA, United States; ^5^ Department of Parasitology and Mycology, Faculty of Pharmacy, Félix Houphouët-Boigny University, Abidjan, Côte d’Ivoire

**Keywords:** ****Fc gamma receptors, polymorphism, malaria, immunoglobulin G, susceptibility

## Abstract

Repeated infections by *Plasmodium falciparum* result in a humoral response that could reduce disease symptoms and prevent the development of clinical malaria. The principal mechanism underlying this humoral response is that immunoglobulin G (IgG) binds directly to the parasites, thus causing their neutralization. However, the action of antibodies alone is not always sufficient to eliminate pathogens from an organism. One key element involved in the recognition of IgG that plays a crucial role in the destruction of the parasites responsible for spreading malaria is the family of Fc gamma receptors. These receptors are expressed on the surface of immune cells. Several polymorphisms have been detected in the genes encoding these receptors, associated with susceptibility or resistance to malaria in different populations. In this review, we describe identified polymorphisms within the family of Fc gamma receptors and the impact of these variations on the response of a host to infection as well as provide new perspectives for the design of an effective vaccine for malaria.

## Introduction

Despite the progress made against malaria, this disease remains a major public health concern, particularly in sub-Saharan Africa (sSA) (1). Indeed, 405,000 malaria-related deaths were reported in 2018, with 94% of them in sSA (2). In malaria-endemic regions, there are populations that present some resistance to the disease (3). In fact, *Plasmodium falciparum*, the main malaria parasite in sSA, is considered to be one of the most important evolutionary forces causing the appearance of several alleles giving the host some protection (4). Natural selection is a process explaining how individuals of a species living in a given environment will tend to exhibit a certain suitability for that environment (5). This principle is believed to be the cause of a larger proportion of individuals with the βS allele in sSA compared to those living in Europe. Indeed, although this allele in homozygotes is associated with hemolytic anemia as well as unpredictable episodes of pain and generalized organ damage, all of which could be life-threatening for the carrier, it does appear in heterozygous individuals to be associated with a 90% greater chance of not developing severe malaria (6).

Understanding the mechanism behind the low susceptibility levels of particular individuals to malaria infection could allow us to develop new tools for controlling such infection. Several association studies have revealed human genes that can give populations some resistance to malaria (7). Such has been the case for Fc gamma receptor (*FCGR*) genes. These genes are in major part localized in an area that is peripheral to the centromere, specifically in the q23 area of chromosome 1 (8). They constitute, therefore, a cluster encoding a number of proteins involved in recognizing the Fc domains of antibodies that bind pathogenic antigens (9). The implications of specific gene clusters for innate and adaptive immunity are of particular interest for those developing new strategies to control disease.

However, the polymorphisms detected within the FCGR genes have been associated with either susceptibility or resistance to malaria. Indeed, the selection phenomenon induced by a pathogen does not always result in the elimination of alleles conferring susceptibility to the host but very often leads to the maintenance of resistance and susceptibility alleles (10). This process leads, therefore, to genetic variability in the population affected by the phenomenon known as balancing selection. This phenomenon may be the source of the difficulty in establishing, in classical association studies, the real influence of a mutation or an allele on the response to malaria (11). Thus, the same allele could be associated with susceptibility in some cases and resistance in others depending on whether or not another allele having an impact on malaria response is present (12).

The United States Food and Drug Administration first authorized the use of antibodies for certain diseases, including cancer, in 1986 (13). Several antibodies have been tested in clinical trials in an effort to combat human immunodeficiency virus (HIV) infection (14) and no antibodies have been approved for the treatment of malaria treatment as of yet (15). Recent studies, each performed in an animal model, showed that the passive administration of anti-tumor antibodies could induce long-term protection *via* a mechanism involving the maturation of dendritic cells and the presentation of tumor antigens to T cells (16). If successfully applied to humans, this approach could improve the efficacy of candidate vaccines for the prevention of malaria. It is therefore necessary to identify polymorphisms in *FCGR* genes that are associated with susceptibility and resistance to malaria in endemic areas.

In this review, we describe the known polymorphisms within the FCGR family of genes and the impact of these variations on the response of the host to infection, as well as provide new perspectives for the design of an effective vaccine against malaria.

## *FCGR* Genes and Their Evolution

The *FCGR* genes are known to form a cluster on human chromosome 1 (17). These genes code for glycoprotein receptors on the surface of immune cells including B cells, macrophages, natural killer (NK) cells and dendritic cells (DCs) (9). Previous research has identified three classes of Fc receptors in humans, including FcγRI, FcγRII and FcγRIII, with each class containing a range of different isoforms. The FcγRI class features the FcγRIA, FcγRIB and FcγRIC isoforms, while the FcγRII class features the FcγRIIA, FcγRIIB and FcγRIIC isoforms. The FcγRIII class features the FcγRIIIA and FcγRIIIB isoforms (9).

The family of *FCGR* genes as described by Hargreaves et al. (18) first appeared millions of years ago in our common ancestor with bony fish. *FCGR2* and *FCGR3* have been hypothesized to be the first genes to appear on the long arm of chromosome 1 (band q23-24) (19, 20). These genes subsequently experienced duplications leading to new isoforms that were initially identical but subsequently diverged, thus giving rise to the *FCGR2A* and *FCGR2B* genes prior to the process of divergence that ultimately led to primates (21). In contrast, the *FCGR3A* and *FCGR3B* genes are thought to have been generated during the divergence of humans and chimpanzees approximately 6 million years ago (22). Later, the *FCGR3B* gene experienced structural changes in its promoter and transmembrane regions (22, 23), thus resulting in copy number variations that are characteristic of this gene. The *FCGR2C* gene may have formed as a result of an unequal crossing over of *FCGR2A* and *FCGR2B* genes (21). The *FCGR1* gene first appeared after the *FCGR2* and *FCGR3* genes; the exact period of this event remains unknown although a previous study did show this gene to be absent in the genome of the opossum (19, 20). Maresco et al. (24) further suggested that the ancestor of the *FCGR1* genes later experienced a duplication that led to the three isoforms that exist to this day, namely *FCGR1A*, *FCGR1B* and *FCGR1C*. The same authors also hypothesized that an inversion may have initially located *FCGR1B* in the centromeric area (1p12 area of the chromosome) and later led to the *FCGR1A* and *FCGR1C* isoforms locating in the 1q21 area of chromosome 1.

## The Structure and Function of Fc GAMMA Receptors

Fc gamma receptors can be structurally considered either as activating receptors (such as FcγRI, FcγRIIA, FcγRIIC and FcγRIIIA) that each have an immunoreceptor tyrosine-based activation motif (ITAM) or inhibitory receptor (FcγRIIB) that contain an immunoreceptor tyrosine-based inhibitory motif (ITIM) (25). These intracytoplasmic regions can either be located on a transducing chain (as is the case of the FcγRI and FcγRIIIA receptors) or they may represent an integral component of the receptors (as is the case of FcγRIIA and FcγRIIC). The FcγRIIIB receptor, while it interacts with other receptors, is the only receptor that does not adopt a mechanism involving signal transduction. Receptors of the FcγRI family are expressed by monocytes, macrophages, neutrophils and dendritic cells. The FcγRIIA receptor is expressed by the same cells as the FcγRIIB receptor and can also directly activate blood platelets. In addition, the FcγRIIC and FcγRIIIA receptors are able to activate NK cells while FcγRIIIB can activate neutrophils and eosinophils (26).

The receptor activation process begins after the antibodies that opsonize parasites are recognized by a specific receptor. This recognition results in an aggregation of the activating receptors that bind the pathogens. This process is then followed a phosphorylation of the two tyrosine (Y) residues of ITAM (the consensus sequence of ITAM motif being **Y**xx[L/I]x(6−8)**Y**xx[L/I]; with x representing any amino acid residue) by Src family tyrosine protein kinases (27, 28). This phosphorylation permits the recruitment of two Src-homology 2 (SH2) domains of the Syk tyrosine kinase in the docking sites formed by both phosphotyrosines and the space between them (29–31). Activated receptors are involved in a wide range of activities, including phagocytosis, the respiratory burst, the production of cytokines by macrophages and DCs, antibody-dependent cell cytotoxicity (ADCC), and the degranulation of neutrophils and NK cells (32, 33). However, it has been observed in some specific circumstances that the ITAM motif responsible for activating the receptors could exhibit inhibitory functions, denoted as ITAMi (ITAM-mediated inhibitory signal) (34). This change is due to a particular reaction in which there is phosphorylation of only one of the two tyrosine residues of the receptor in the presence of the kinase Src (35). Two juxtaposed receptors carrying an ITAM motif and each motif having only one of their tyrosine residues phosphorylated would in this case be able to bind tandem SH2 domains of the SH2-domain containing protein tyrosine phosphatase 1 (SHP-1) (34, 35). A binding comparable to that formed between two ITIM motifs and SHP1 is obtained—causing Lyn phosphorylation of tyrosine at position 536 and regulating SHP-1 phosphatase and inhibiting cell activation (36). This particular inhibition of ITAM-like receptors would have as an effect reduction of inflammation in animals and would serve as a basic mechanism for controlling the activation of both FcγRIIA and FcγRIIIA receptors (35, 37).

Another mechanism for the inhibition of the activating receptor involves the inhibitory receptor (FcγRIIB), which presents an ITIM motif with only one tyrosine residue [I/V/L/S]x**Y**xx[L/V] (27). This receptor is highly expressed in B cells and can inhibit the activation of signals induced by B-cell receptors (BCRs) and hence regulate their effects (38). During this process, the ITIM motif, characterized by the presence of a single tyrosine, is phosphorylated by the protein tyrosine kinase (PTK) Src which allows the recruitment of the two SH2 domains from the proteins inositol phosphatase 1 and 2 (SHIP-1 and SHIP-2) (27). This recruitment leads to a cascade of events, in turn leading to inhibition of Ig-calcium flux (39), the dephosphorylation of the activating receptor, and as a result to its inactivation (40). Thus, the inhibitory action of FcγRIIB receptors requires the co-ligation of an inhibitory and activating receptor *via* an immune complex (27). However, although the scientific community entirely accepts the idea that the FcγRIIB receptor is able to inhibit activating receptors of the BCR type, some studies question the ability of FcγRIIB to inhibit phagocytic activity of myeloid cells (40). Indeed, by an mRNA splicing of exon C1 leading to the form of FcγRIIB called FcγRIIB2 instead of the form FcγRIIB1 of B cells (34), the FcγRIIB receptor can be also expressed in monocytes, macrophages, neutrophils, and dendritic cells. During phagocytosis induced in transfected Chinese hamster fibroblast cells, the inhibitory FcγRIIB receptors have been shown to be at least half as numerous as the activating FcγRIIA receptors (41). However, these authors showed that an equi-proportional amount of the two types of receptors would be required to allow inhibition. The low number of the inhibitory FcγRIIB compared to the activating FcγRIIA receptors could thus affect the ability of FcγRIIB to inhibit the activity of FcγRIIA receptors (41). Do these observations indicate that the preferential mechanism of inhibition of FcγRIIA is that involving the ITAMi? Perhaps, but it could be noticed that some mutations suppressing the inhibitor receptor have been associated with increasing macrophage phagocytosis of *Plasmodium* (42). Moreover, the mechanism called “inside-out control” and well reviewed by Koenderman et al. (43) could may be explained how FcγRIIB apparently in low number succeed to inhibit FcγRIIA’s activity. Indeed, by modulating the affinity, i.e., the strength of the receptor for its ligand, modulating the valency or the engagement of multiple receptors, modulating the interaction with the signal chains, the other receptors, or their localization in the various membranes, it is possible to regulate the function of the receptors. Different mechanisms are therefore involved. FcγRI receptors, which normally bind monomeric antibodies, can be activated and bind immune complexes by the action of the phosphatase PP2A, an enzyme involved in the dephosphorylation of FcγRI receptors, and probably by the lateral movement of the receptor in the membrane (44). For FcγRIIA receptors, the engagement of mitogen-activated protein kinase kinases (MEK-MAPK) has been indicated to be involved in the activation of the receptor (45) as far as the level of expression of the tail of the receptor (46, 47). The expression of the FcγRIIIA receptor has been indicated to be influenced by the glycosylation. The review by Koenderman et al. (43) mentioned that an ectopically expressing tail-less version of FcγRIIB could weaken the signal response. Again, some studies have shown that a mutation in FCGR2C genes to a stop codon leads to the expression of FcγRIIB in NK cells with consequences of inhibiting its function (34, 48). Finally, the authors mentioned that “inside-out control’’ of Fcγ receptors could also be achieved by receptors others than Fcγ receptors (toll-like receptor, cytokine/chemokine receptors, glucan receptors) (49–51).

## Methods for Identifying Polymorphisms in *FCGR* Genes

Several methods have been used to investigate the association between polymorphisms in the *FCGR* genes and the immune response to malaria. This topic was recently reviewed by Hargreaves et al. (18). The simplest approach uses the polymerase chain reaction (PCR) in combination with restriction enzyme digestion; this process is known as allele-specific restriction enzyme digestion (ASRED) and is able to differentiate alleles and single nucleotide polymorphisms (SNPs) or single nucleotide variants (SNVs). A SNP is a substitution occurring in at least 1% in the general population and an SNV is a variation of a single nucleotide without limitation of frequency (52–54). The advantages of this method are that it is relatively inexpensive and easy to perform (55). However, the high similarity of genes within the same specific cluster can make it very difficult to design primers that are able to amplify specific regions of the target DNA.

An alternative strategy uses the TaqMan assay (Life Technologies, Paisley, UK) to identify SNPs within a family cluster (18). This method uses a probe with a fluorescent dye on the 5´-end, while a nonfluorescent quencher on the 3´-end inhibits the fluorescence. The probe, once added to a sample of human DNA, would hybridize to the target sequence, if present. During the elongation step of the PCR reaction, the *Taq* polymerase cleaves the 5´-end of the probe, thus liberating the dye from the quencher and fluorescing light that can be detected, thus confirming the efficacy of the reaction. The TaqMan assay has been used successfully to determine the distribution of *FCGR2B-*rs1050519, *FCGR2C-*rs3933769 and *FCGR3A-*rs396991 SNPs in sympatric ethnic groups from Mali (56). This method has also been used to identify insertions and deletions in sequences of DNA. For example, a TaqMan assay was used to identify in the promoter of the *NF-k B1* gene a 94-base-pair insertion/deletion polymorphism (rs28362491) that was associated with bladder cancer in the Chinese population (57). This method has also been used to determine the copy number variation (CNV) values of genes; for example, Qi et al. (58) using TaqMan assays observed that low CNVs of *FCGR3A* and *FCGR3B* in Chinese patients were associated with systemic lupus erythematosus (SLE). The TaqMan assay clearly has many advantages in that it is relatively inexpensive, uses low quantities of DNA and can be used for a variety of applications. However, the main disadvantage of this method is that the amplicons it produces are very short. This disadvantage represents a serious problem when the target region of the *FCGR* gene shares a broad homologous domain with another gene within the same cluster, as is the case for both the *FCGR2B* and *FCGR2C* genes (18, 59).

The multiplex ligation-dependent probe amplification assay (MLPA) is similar to the TaqMan assay in that it can detect the CNV of a genomic sequence by using a specific probe. However, in the MLPA, the probe contains two separate parts that target the same specific sequence of a gene. Once bound to the target sequence, both parts of the probe are ligated and are amplified by PCR using primers that are located at each end of the probe; CNV can then be quantified from the resultant fluorescence. This method was previously used to investigate variability in all genes located within a specific cluster of Black and Caucasian subjects in the 1000 genomes project (60). However, this method tends to be expensive, because it relies on the co-amplification of multiple genes of interest in one reaction.

Pyrosequencing is based on the introduction of a known nucleotide into the sequencing mix. This known nucleotide is subsequently incorporated into the DNA fragment being sequenced when it encounters a complementary nucleotide in this DNA fragment. This incorporation results in the release of pyrophosphate, which in the presence of firefly luciferase enzyme is converted into adenosine triphosphate (ATP). Since this reaction is luminescent, it can be readily detected. This method is advantageous because it allows for the study of two sequences with high levels of similarity. However, the major drawback of pyrosequencing is that it is not efficient in homopolymeric regions, i.e., genomic sequences that feature the same base sequence but are of different sizes (18, 61).

Next-generation sequencing (NGS) also represents a useful technology for sequencing and differentiating between FcγR gene clusters. Pacific Biosciences, Inc, of California, USA (PacBio) has developed a new technology referred to as Single Molecule, Real-Time (SMRT) Sequencing. This method is a parallelized single-molecule DNA sequencing method and is based on the conversion of a double-stranded piece of DNA into one circular strand by ligating hairpin adaptors at the ends of the sequence. This method was previously used by Hargreaves *et al.* to sequence the *FCGR2B* gene in a single 14 kb fragment, but has also been shown to support the sequencing of fragments up to 60 kb in length in one single fragment (18, 62). However, SMRT is associated with a high error rate (13%) and is a low throughput method. In addition, SMRT sequencing tends to be more expensive than other forms of NGS technology.

## Polymorphisms in *FCGR* Genes and Their Impact on the Response of a Host to Malaria

Two classes of FcγRs can be identified on the basis of their affinities for monomeric IgG. The high-affinity class is only composed of the FcγRI receptor, while the class of low-affinity receptors includes both FcγRII (A, B, and C) and FcγRIII (A and B). Because the FcγRI receptor binds with high affinity to monomeric IgG, previous researchers postulated that the receptor might not be able to bind to other immune complexes (63) and has not, therefore, been studied extensively. However, recent studies have highlighted the importance of the FcγRI receptor in immunological responses. In this part of our review, we present an overview of our current understanding of the genes that encode both classes of receptors and their relative involvements in the response to malaria.

### Genes Encoding the High-Affinity Receptor FcγR: The *FCGR1* Genes

The human FcγRI receptor is encoded by three highly homologous genes that are located on band q21 (*FCGR1A* and *FCGR1C*) and band p12 of chromosome 1 (*FCGR1B*) (24). The *FCGR1A* gene is the only one that encodes a complete 72 kDa cell surface receptor protein featuring three extracellular immunoglobulin-like domains (D1, D2, and D3) (64, 65). The D3 domain, which is absent in low-affinity receptors, is believed to provide the high-affinity binding capacity that enables FcγRI receptors to bind to monomeric antibodies (66, 67). However, the two domains (D1 and D2) of the FcγRI receptor are similar to those found in the low-affinity receptors and could allow the receptor to bind immune complexes. The *FCGR1B* and *FCGR1C* genes are pseudogenes that generally encode non-functional proteins (68, 69). But, a splice variant that lacks exon 5 (the region of the gene responsible for expressing the D3 domain) enable the pseudogene *FCGR1B* (when inserted into mouse DNA) to encode a receptor capable of binding aggregated antibodies (70).

Described by some authors as less polymorphic than the other *FCGR* genes, the *FCGR1* gene is associated with five SNVs (71). Three of these SNVs are non-synonymous (rs7531523, *V39I*; rs12078005, *I301M*; and rs142350980, *I338T*); the other two are nonsense (rs74315310, *R92X;* and rs1338887, *Q224X)*. In a recent study, Brandsma et al. (71) investigated the effects of the three non-synonymous SNVs on the function of the *FCGR1A* receptor in murine models and observed in each case a clear breakdown in the immune response. The first SNV, rs7531523 (*V39I*) reduced the ability of the FcγRI receptor to bind immune complexes. The other two SNVs, rs12078005 (I*1301M*) and rs1050208 (*I338T*), reduced FcγR1 signaling. These authors also noticed that the both SNVs had a low occurrence rate in Dutch individuals. Collectively, this information leads us to hypothesize that the deleterious immune response caused by these variations may have contributed to negative selection during evolution and therefore highlights the importance of the FcγRI receptor in controlling diseases such as malaria.

Indeed, Loughland et al. (72) observed an important expression of FcγRI in intermediate monocytes involved in antibody-mediated phagocytosis of *P. falciparum* (73). These monocytes are much more important in Fulani people than in Mossi people, since the Mossi are more susceptible to malaria (3). It seems therefore, when considering the study of Loughand et al. (72), that the immune system has a tendency to solicit FcγRI in intermediate monocytes to contain infection, as their results confirmed an increase of the expression of intermediate monocytes and FcγRI with the evolution of the disease. However Dobbs et al. (72) observed an association of overexpression of FcγRI receptor in monocytes during acute malaria with a reduction of phagocytosis in comparison with the level observed 6 weeks after the treatment (74). According to the authors, this association could be due to the effect of the inhibitory receptor FcγRIIB and the recruitment of the SHIP phosphatase having a consequence of negatively regulating phagocytosis. But this explanation has not been proved. In another study, McIntosh et al. (75) reported that the transgenic incorporation of the human FcγRI receptor into a mouse model allowed these mice to clear malaria parasites in the presence of IgG1 against merozoite surface protein 19 or anti-MSP19. However, as suggested by Brandsma et al. (71) there must be redundant properties of the activities of the FcγR receptors. Indeed, the occurrence of an SNV in the first extracellular domain-encoding region of the FcγR receptor was shown to lead to a complete loss of the receptor expression without any apparent consequence in the ability of subjects to control infection (48, 68). More studies will be needed to understand the influence of variations in the gene FCGR1 on responses to malaria.

### Genes Encoding Low-Affinity Receptors

**Figure 1:**
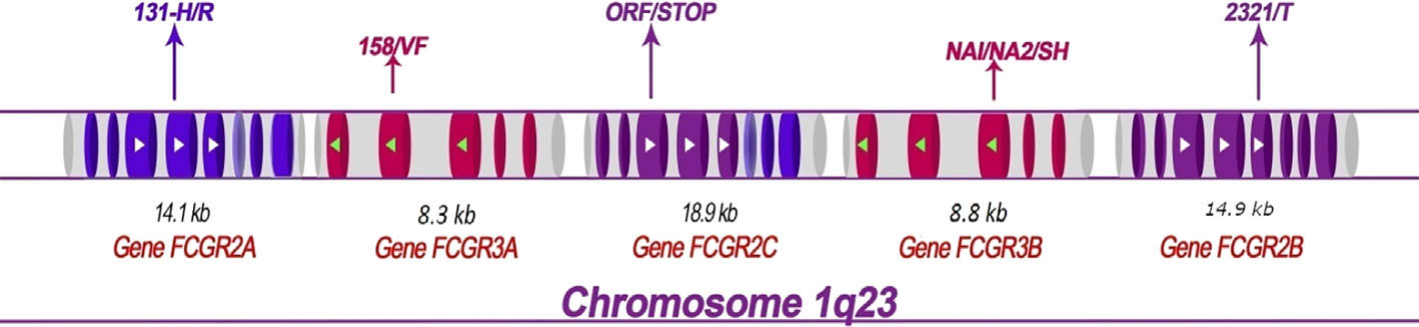
Structural map showing the *FCGR2* and *FCGR3* genes on human chromosome 1q23 and depicting the principal polymorphisms associated with malaria/susceptibility. The coding regions for the *FCGR2* and *FCGR3* genes are shown in color while the non-coding regions are shown in grey. The 6th exon of the FCGR2C gene is spliced ​​into the FCGR2A and 2C genes (lighter shade). Important SNPs that are associated with disease susceptibility/resistance, along with their patterns of localization, are indicated by arrows. For example, the *FCGR2A*-131H/R single nucleotide polymorphism occurs in the fourth exon of the gene *FCGR2A*. The *FCGR* genes show high levels of sequence homology. The coding regions that exhibit similar sequences are shown in the same color (note that the sequences of the *FCGR3A* and *FCGR3B* genes are identical). The directional aspects of different genes are specified by the tips of the arrows; white indicates left to right while green indicates right to left. The first exon of the FCGR2A gene is thus at the left end and the last exon of the FCGR2B gene at the right end of the figure.

Five *FCGR* genes encode low-affinity receptors for monomeric IgG: *FCGR2A*, *FCGR2B*, *FCGR3A*, and *FCGR3B*. These genes have lengths ranging from 8.3 kb (for *FCGR3A*) to 18.9 kb (for *FCGR2C*) and each gene contains multiple exons (**Figure 1**). Several SNPs have been reported in low-affinity *FCGR* genes and associated with the host response to malaria (**Table 1**).

**Table 1:** Variations in FCGR genes associated with susceptibility or resistance to malaria.

Gene	RefSNP	Variations	Effect	Outcome	Malaria	Country	Population	Statistic	P-value	Authors
FCGR2A	rs1801274	RR131 homozygote	FcγRIIA-RR131 bind IgG2 less efficiently than FcγRIIA-HH131	Protective	HP	Kenya	High vs. low risk group	17% vs. 34%	0.0021	(76)
	rs1801274	HH131 homozygote	HH131 genotype have a greater quantitative activation of the innate immune system can be achieved by a broader repertoire of antibodies, increasing the risk of immunopathology and disease	At risk	SM	Gambia	Severe vs. control	26.3% vs. 21.7%	0.034	(77)
	rs1801274	H131 allele	FcγRIIA -131H, the only human FcγR capable of binding IgG2 efficiently and that its polymorphism affects the regulation of the production or turnover of the IgG subclass in humans	Protective	CLM	Asia, Africa	Cases vs. controls	8,688 vs. 5,706 (number of alleles)	0.009	(78)
FCGR2B	rs1050501	TT232 homozygote	Removed expression of receptor Suppress inhibitory effect increase macrophage phagocytose Receptor FcγIIB deficiency associated to less parasitemia in mice (79)	Protective	SM	Kenya	Severe vs. control	07.84% vs. 04.59%	7.1 10^-5^	(42)
FCGR2C	CNV	Higher copies	Altering the balance of activating and inhibitory FcγR on immune cells (80) Prevent excessive immune responses by restricting number of receptors on cell surface	At risk	SM	Kenya	Mild vs. severe	5.3 ± 0.9 vs. 4.3 ± 0.8 (mean copies)	< 0.0001	(81)
	Intron rs3933769	T allele	To be determined	Protective	HP	Mali	Fulani vs. Dogon	76.19% vs. 36.87%	< 0.0001	(82)
FCGR3A	rs396991/ rs5743836	158V/1237T(TLR9) haplotype	Increase production of IFN-γ	At risk	SMA	Kenya	Non SMA vs. SMA	20.9% vs. 33.3%	0.009	(12)
FCGR3B	rs403016/ rs447536/ rs448740/ rs428888/ rs2290834	NA2*03 (N82D)	Lead to one glycosylation site	At risk	CLM	Ghana	cases vs. controls	12.5% vs. 04.1%	0.0092	(83)
	rs5030738	SH allotype	May influence ligand epitope Associated with high FcγRIIIB expression level	Protective	CLM	Ghana	cases vs. controls	06.7% vs. 20.1%	0.049	(83)
	CNV	CNV > 3 copies	Overactivation of immune cells Create inflammatory disorders and exacerbate symptoms	At risk	SM	Gabon	severe vs. mild	2.8 ± 0.7 vs. 2.3 ± 0.6 (mean copies)	< 0.0001	(81)
FCGR2A FCGR3B	rs1801274/ rs403016/ rs447536/ rs448740/ rs428888/ rs2290834	H131/NA2 haplotype	To be determined	At risk	SMA	Kenya	131H/NA2 vs. non-131H/NA2	RR=1.47	0.020	(84)
	rs1801274 rs403016/ rs447536/ rs448740/ rs428888/ rs2290834	H131/NA2 haplotype	Neutrophils with the FcγRIIIB-NA2/NA2 genotype show lower activation of FcγRIIA-mediated phagocytosis than those with the FcγRIIIB-NA1/NA1 genotype	At risk	CM	Kenya	cerebral vs. non-cerebral	43.9% vs. 36.3%	0.012	(85)
FCGR2A FCGR3A FCGR3B	rs1801274/rs396991/ rs403016/ rs447536/ rs448740/ rs428888/ rs2290834	131H/158F/NA1 haplotype	May be low binding of cytophilic antibodies due to the diluting effect of 158F allele	At risk	HP	Kenya	131H/158F/NA1 vs. non-131H/158F/NA1	4.37 vs. 4.12 (mean number of carriers)	0.009	(56)
	rs1801274/rs396991/ rs403016/ rs447536/ rs448740/ rs428888/ rs2290834	131R/158F/NA2	May be reduced cross-linking in neutrophils, hence low phagocytic activity, reduced antibody dependent respiratory burst	At risk	SMA	Kenya	SMA vs. non-SMA	69.3% vs. 57.5%	0.036	(56)

RefSNP accession number of SNP, Variations, SNP, allele, allotype, haplotype, genotype associated with malaria susceptibility or resistance, Effect, impact of the variation in immune response as mentioned by the authors of the study (see Authors column): or other authors (in parentheses), Outcome, impact of the variation on the malaria response of the population; Malaria, form of the disease impacted by the variation; HP, hyperparasitemia; SM, severe malaria; CM, cerebral malaria; CLM, clinical malaria; SMA, severe malaria anemia; Country, country where the variation was studied; Population, population in which the statistics [mean, frequency, relative risks (RR), number of gene copies (cp)] of each variation were compiled, p-value, statistical significance of the difference between statistics variations (threshold of significance being less than 0.05); Authors, reference of authors of the articles that mentioned the variation.

#### The *FCGR2A* Gene

The *FCGR2A* gene harbors an important nucleotide substitution (G to A) that changes the amino acid residue at position 131 of the distal immunoglobulin domain; this substitution is known as *H131R* or SNP rs1801274. Depending on whether or not the guanine (G) base is present in the DNA sequence, the corresponding amino acid residue could either be an arginine (allele *R131*) or a histidine (allele *H131*). The receptors encoded by allele *H131* have been shown to have better affinities for IgG2 than those encoded by allele *R131* (21, 86). The FcγRIIA-H131 receptor has been shown to be capable of binding three different immunoglobulins (IgG1, 2, and 3) more efficiently than has the FcγRIIA-R131 variant. This broad coverage increases the phagocytic capacity of the *FcγRIIA-H131* receptors (87). Consequently, researchers have investigated the association between *H131R* and its potential ability to provide protection against several diseases (88, 89). The first indication that the *H131* allele could be involved in providing protection against an infectious disease like malaria came from observing differences in allele frequencies when comparing Asian populations (in which malaria was the primary cause of 19,700 deaths in 2017) and European populations (90). In field studies, 28% of Caucasians (91) were shown to be homozygous for *H131* while 71.5% of Asians were shown to be homozygous (92). The *H131* allele is therefore more prevalent in Asian populations than in populations like Caucasians who are not subjected to *P. falciparum* selective pressure.

The occurrences of the *H131* allele in various African peoples have also been studied. Indeed, the frequencies of the *H131* and *R131* alleles vary according to ethnic grouping in Africa. Three different studies showed similar distributions of *H131* homozygotes (27.37% out of 274 subjects, 32% out of 97 subjects, and 20.45% out of 88 subjects) in Luo and Luhya populations of Kenya and a Yoruba population of Nigeria (56, 60). However, different distributions of the genotype for this SNP were found for different ethnic groups from Ghana (p=0.036), with the frequency of subjects who were homozygous for *R131* being higher for the Ga-Adangbe ethnic group than for the Akan, Hausa and Fulani ethnic groups (p=0.01347; Chi square test performed by us and R according to the results given by the authors in the Supplementary Table in SI) (93). In this way, it has been observed that in Mali and Eastern Sudan, the Fulani, who are less susceptible to malaria than other sympatric ethnic groups (94), exhibited a greater frequency of the *H131* allele than did a non-Fulani group (95, 96).

Association studies were also carried out to assess any possible influence of SNPs on the control of malaria; these studies led to inconclusive findings. While a few of the studies suggested an association between the *H131* allele and a protective effect against malaria (95, 96), others have reported an association between this allele and the occurrence of severe or cerebral malaria (77, 85, 96). The *R131* allele has also been associated with low parasitemia in western Kenya (76). Finally, a meta-analysis of *H131* and *R131* suggested a strong association between the *FcγRIIA*-*H131* allele and protection against malaria in Asian and African populations (78). The authors of this meta-analysis attributed the conflicting results in the existing literature to low statistical power, racial differences and inadequate study design. It is also possible that these contradictory results may have been due to hypomethylation in the promoter of the *FCGR2A* gene, as observed in Taiwanese people (97). Hypomethylation can lead to overexpression of a gene consequently, it is plausible that a certain level of gene expression might be required to observe an effect on malaria. It is also possible that a high level of gene expression may lead to an inflammatory response that causes a transformation from a mild form of malaria to a more severe form. Finally, these contradictions could be simply due to the action of balancing selection, keeping the non-advantageous form of the gene for malaria control in some populations because it presents a certain advantage for controlling other diseases, as suggested by Duxbury et al. (10). So, the future studies should take into account these different hypotheses to best understand the implication the SNPs in the control of malaria.

#### The *FCGR2B* Gene

SNP rs1050501 in the *FCGR2B* gene may be under the influence of evolutionary pressure caused by malaria (98). This SNP leads to the replacement of isoleucine by threonine at position 232 in the transmembrane domain of the protein encoded by the *FCGR2B* gene. The presence of threonine abrogates the inhibitory function of the receptor, thus leading to the increased phagocytosis of parasites by macrophages (79). Mice deficient in the receptor FcγRIIb have shown on average lower levels of parasitemia and less severe anemia than have non-deficient mice (79). In humans, a higher frequency of the *FCGR2B-*T232 allele has been observed in Asian and African populations than in the Caucasian population (79, 99, 100). In addition, a cohort study involving Kenyan children revealed that those homozygous for *FCGR2B-*T232 exhibited lower levels of susceptibility to severe malaria (101).

The *FCGR2B* gene also presents with a haplotype in the promoter region (-*386C: -120A*) including two SNVs, at codons 386 and 120, responsible for replacing cysteine and alanine with glycine and threonine, respectively (102). This haplotype has been associated with an increased level of gene expression in monocytes, neutrophils and dendritic cells (103, 104). In contrast, Blank et al. (105) showed a relatively high susceptibility of European-Americans homozygous for the first SNV (-*386C/C* or -*343C/C* if considering the start of transcription), to systemic lupus erythematosus (SLE), an autoimmune disease. These authors also observed that these individuals had lower expression levels of FcγRIIb receptors on the surface of activated B cells. Investigating the impact of this SNP in the African population would therefore be expected to enhance our understanding of the precise role of the *FCGR* gene in malaria susceptibility.

#### The *FCG2C* Gene

The *FCGR2C* gene is often referred to as a chimeric or pseudogene gene because it does not always encode a protein; this characteristic is due to the presence of the mutation *Q13X* at position 13 in exon 3 of this gene (45, 99). Consequently, the expression of the *FCGR2C* gene by NK cells is in fact an open reading frame (ORF) due to the substitution of a thymine residue by cytosine, thus resulting in the replacement of a stop codon (TAG) with a glutamine residue (CAG) (26). However, this study found that approximately 20% of Caucasians with an *FCGR2C-ORF* genotype did not express the FcγRIIc receptor on the surfaces of their NK cells. Indeed, these authors noted the presence, within the splicing site of intron 7, of a second mutation (G → A) that led to another stop codon, and thus the inability to express FcγRIIc receptors on the surface of NK cells.

In contrast to *FCGR2A* and *FCGR2B*, the *FCGR2C* gene leads to a higher level of protein expression in Caucasians than in Africans (in which the *FCGR2C-ORF* gene is weaker) and Asians (in which the gene is absent) (8, 60). Moreover, van der Heijden et al. (48) mentioned that a nonclassical STOP allele of FCGR2C due to a splice site mutation near exon 7 could promote the expression of FcγRIIb in NK cells. This receptor is not normally detected in these cells. Therefore, the NK cell expression of FcγRIIb has been associated by van der Heijden et al. to an inhibition of the killing activity of *F*cγRIIIa mediated by antibody-dependent cell cytotoxicity. Collectively, this information might suggest that this gene has less influence than the other genes described above on resistance to malaria. This theory is supported by the observation of Gabonese children displaying a higher risk of developing severe malaria when presenting with a high CNV of the *FCGR2C* gene (81). But, the increased number of copies in FCGR2C concerns only the FCGR2C ORF. This means that an increase in the number of copies of FCGR2C would lead to a possible overexpression of the receptor FcγRIIc in NK cells and then overly high immune activation with a consequence of increasing inflammation, resulting in a more severe form of the disease (8, 80).

Existing data indicate that the precise role of this gene is complex. Indeed, an SNP occurring in intron 7 of the *FCGR2C* gene, referred to as rs3933769, is far more common in Fulani subjects from Mali than in Dogon subjects (82). As a distinct ethnic group, the Fulani are known to be less susceptible to malaria than are other sympatric ethnic groups (93). This study demonstrated an association between the mutant allele of this SNP and clearance of parasitemia (82). This could highlight the importance of this particular intron in protecting the Fulani people against malaria. Further studies are needed to elucidate the precise role of the *FCGR2C* gene in the response to malaria.

#### The *FCGR3A* Gene

Two polymorphisms in the *FCGR3A* gene could potentially play a role in providing protection against malaria. The rs396991 polymorphism is a non-synonymous single nucleotide polymorphism from T to G at nucleotide position 559 in the *FCGR3A* gene. This SNP is responsible for substitution of a phenylalanine for a valine amino acid position 158 of the protein excluding signal peptides (*FCGR3A-F/V158*) or at position 176 of the full protein (*FCGR3A-F/V176*) (8). It seems that the wild type (*FCGR3A-158F*) alters the capacity of NK cells to bind the cytophilic antibodies Ig1 and IgG3 (106, 107). Indeed, this allele, combined with *FCGR2A-131R* and *FCGR3B-NA1*, was indicated to be associated with high levels of parasitemia in children from western Kenya, while the *FCGR2A-131R*/*FCGR3A-158F*/*FCGR3B-NA2* haplotype was associated with susceptibility to severe malarial anemia (SMA) (56). In contrast, the *FCGR3A-158V* genotype allows the encoding receptor at the surface of NK cells to bind cytophilic antibodies-coated target cell and therefore the release of cytotoxic effector proteins by NK cells. This activation of NK cells able the death of the target cell (28, 108, 109). However, the influence of SNP *V158F* on the immune response could be affected by the presence of the *-1237T/C* SNP in the *TLR9* promoter gene (thymine base mutated to cytosine at position -1237 of the TLR9 promoter region) (12). Toll-like receptors (TLRs) are proteins belonging to the family of pattern recognition receptors (PRRs). These proteins are localized specifically in antigen-presenting cells and are involved in the detection of pathogenic molecules. In particular, TLR9 is an intracellular TLR that stimulates immune cells and releases interleukin 12, a cytokine involved in the differentiation of naïve T cells from helper T cells but also involved in the production of interferon gamma (IFN-γ) (110). Thus, when associated with *FCGR3A-176V*, the presence of the variant *-1237T* would double the risk of developing severe malaria anemia by increasing the production of IFN-γ (12).

Another important polymorphism in the *FCGR3A* gene is the rs10127939 SNP. This SNP results from the substitution of a T to either a G or A, thus replacing leucine by arginine or histidine at amino acid position 66 in the extracellular domain (111, 112). Some authors have shown the SNP *L66R/H* and the SNP *V176F* to be associated with a reduced cytotoxic response and IgG binding capacity in the NK cells of patients experiencing recurrent infections with Herpes simplex virus, Epstein-Barr virus or Varicella zoster (111). This observation has led to the speculation that both SNPs should lead to increased levels of susceptibility to different infections and autoimmune diseases (109, 112, 113). However, in a study involving 115 Colombians, neither of these SNPs showed any association with the phenomenon of antibody-dependent cell-mediated cytotoxicity (ADCC) in NK cells (111). However, the authors of this study found a linkage disequilibrium between the SNP *L66R/H* and the SNP *V176F* in the *FCGR3A* gene. Furthermore, the 176FF variant appears to result in alterations to the epitope that would normally interact with IgG1 antibodies (111). Further studies should be undertaken to specifically investigate the effect of these SNPs in a population that is endemic for malaria, as no previous research has been attempted in such a population. Pursuing such studies is particularly important considering the presence of an association between lupus nephritis and the *66R/H/L* and *176F* variants in African Americans but not in Caucasians (114). Again, it will also be important to study the impact of CNV of this gene on malaria susceptibility as an CNV higher than 2 increases expression of the receptor (40).

#### The *FCGR3B* Gene

The *FCGR3B* gene codes for a receptor that is expressed on neutrophils. Three isoforms of the FcγRIIIb receptor have been described, each exhibiting a different ability to bind antibodies (115). These allotypes include several human neutrophil antigens, namely HNA-1a (NA1), HNA-1b (NA2), and HNA-1c (SH) (83).

HNA-1a (NA1) and HNA-1b (NA2) are encoded by FCGR3B*1 and FCGR3B*2, respectively (83). Differences between FCGR3B*1 and FCGR3B*2 at five nucleotide positions (c.108C>G; c.114T>C; c.194A>G; c.244A>G; and c.316A>G), which lead to differences at only four different amino acid residues (due to c.114T>C resulting in a synonymous replacement), allow us to distinguish between these two allotypes of the *FCGR3B* gene. This set of differences leads to the presence of two glycosylation sites in the NA2 allotype. It is likely that these glycosylation sites are responsible for the NA1 allotype being more efficient than the NA2 allotype (116). Indeed, compared to the NA2 allotype, the NA1 allotype has a greater ability to facilitate the phagocytosis of opsonized particles by IgG1 and IgG3 (116). Moreover, when combined with the *FCGR2A-H131* allele, the NA2 allotype has been associated with cerebral malaria in Thailand and severe anemia in Kenya (84, 85). Similarly, a variant of the NA2 allotype, NA*203, which has only one glycosylation site, has been associated with susceptibility to malaria in Ghana (83). This variant results in the substitution of asparagine with aspartic acid at position 82 (N82D); and the disruption of receptor function may be due to the loss of a carbohydrate group that is involved in ligand binding affinity and immunogenicity (83, 117, 118). But, it appears also that the final impact of the receptors on immune response would be influenced by glycosylation of IgG depending on whether the glycosylated IgG contains N-glycan (43). Therefore, these inside-out controls of the receptors could be at the origin of discrepancies when trying to assess their role in malaria response.

HNA-1c (SH) is the third most commonly studied allotype of the *FCGR3B* gene. This allotype is encoded by the allele FCG3B*3 (83), which differs from the FCGR3B*2 allele by virtue of an SNP at position c.233C>A (rs5030738) resulting in the replacement of alanine with aspartic acid at position 78. The c.233A allele has already been associated with a protective effect against malaria in Ghanaian children (83). Neutrophils from individuals who have this allele appear, according to the research, to be able to bind malaria IgG antibodies more efficiently than those who express the c.233C allele (119). However, another study involving Gabonese children showed that those having a CNV greater than 3 for *FCGR3B* displayed a significantly higher risk of contracting severe malaria (81). The association between CNV and severe malaria has also been reported for *FCGR2C* genes. Consequently, it appears that an increase in the expression of the *FcγR* gene could lead to the overactivation of immune cells, thus creating inflammatory disorders and exacerbating the symptoms of malaria (81, 120, 121).

## Fcγ Receptors and the Development of New Vaccine Strategies

Chloroquine resistance, which began in Southeast Asia in the 1960s and then spread to Africa, increasing the number of deaths from malaria, has led to the adoption in several countries since 2005 of artemisinin combination-based therapies (ACTs). However, resistance to artemisinin was reported in Southeast Asia (2, 122). If this resistance were to spread around the world as did chloroquine resistance, the consequences could be devastating for the countries affected. Also, it is more important than ever to think of new strategies for combating malaria. In particular, the development of an effective vaccine would make it possible to curb the phenomenon of selection of resistant strains, which is very common with antimalarial drugs because of drug pressure. But research to this effect has been complicated by the variability of the antigens carried by the parasite. Thus, the currently most advanced candidate vaccine allows only partial short-term protection against malaria (50% reduction in malaria incidence over 1 year) (123).

Therefore, finding a methodology to boost candidate vaccine antigens would be a welcome solution to the challenges of reaching the goal of eliminating malaria. From this perspective, therapeutic antibodies could offer an avenue. Indeed, Douglas et al. (124) showed that the transfer of neutralizing antiplasmodial antibodies to primates induces protection against *Plasmodium falciparum* without resorting to treatment. While the quantities used to achieve this test result were large, the study clearly offered a plausible solution. But a limitation of this study was the non-integration of the pathogen destruction mechanism by the activation of complement and the opsonization of particles. As shown by Koenderman et al. (43), it may be possible to reduce the therapeutic doses of antibodies by impeding the inside-out control of FcγR.

Another study identified monoclonal antibodies capable of recognizing different strains of parasites from RIFIN (repetitive interspersed family) proteins which are antigens expressed on the surface of infected red blood cells (125). The variable domains of such antibodies could, according to Shi et al. (15), be used to design a particular antibodies called bispecific antibodies capable of targeting the antigens of interest. These antibodies present another variable domains of the light and heavy chain (VL,VH) (126) that could also be designed to recognize specifically a given FcγR receptor (127, 128). Additionally, these antibodies can also be designed to target the FcyR receptors located on memory T cells or receptors involved in stimulating the presentation of antigens to DCs like FcγRIIA. These DCs in turn activate the memory T cells (16). According to Anania et al. (34), the use of anti-CD3/CD28 could directly stimulate the intracellular and surface expression of FcγRIIA receptors. Thus, a new vaccine strategy against malaria might involve combining both antibodies not linked to the antigens, thereby making it possible for them to not only serve as therapy for an infection in progress but also to induce a long-term response, a “vaccine” effect of antibodies, when they engage memory T cells. These antibodies could boost the efficiency of the candidate antigen vaccines to which they would be linked (15). Therapeutic antibodies would thus serve as a vehicle for candidate antigen vaccines instead of the use of traditional adjuvants.

But one of the obstacles to using therapeutic antibodies is their cost of manufacture, which would not be profitable since these therapeutics would be intended for poor countries where malaria is prevalent. However, as Shi et al. (15) showed, bispecific antibodies can be produced simply and economically by carrying out chemical conjugation or recombinant expression systems.

Hart et al. (129) indicated that certain NK cells that play an important role in malaria resistance do not present Fcγ receptors but rather NKG2C (two C-type lectin protein expressed by NK cells) receptors in individuals who at some point in their lives had been infected with cytomegalovirus (representing 81.8% in Africa, (130). Taking this study into account, one could imagine that the antibodies not linked to human complexes of the new vaccine against malaria could be specifically directed against these cells.

Combined, these studies show the seemingly endless possibilities for developing new vaccine strategies against malaria.

## Conclusions and Perspectives

FcγR receptors modulate the immune response by interacting with the Fc domain of an antibody. This interaction stimulates the activation of innate immune cells and the production of antibodies and is also known to influence the activities of antigen-presenting cells. Therefore, by enhancing our understanding of the functional activity of the FcγR receptors, we may be able to develop new strategies for vaccination. Cancer patients have been screened for polymorphisms in the *FCGR* genes that encode for FcγR receptors in order to design antibodies in which the Fc domain induces better recognition by immune cells. Individuals with the *FCGR3A-158VV* genotype have been shown to exhibit better responses to therapeutic antibodies, including rituximab (131). This new concept allows for individualized treatments of patients and leads to better results.

Despite the success associated with passive immunization strategies involving anti-malaria antibodies (15), such techniques have not yet been applied for the clinical treatment of malaria. However, new engineering approaches now allow us to design antibodies against specific epitopes of plasmodial surface antigens (15). In addition, a new form of synthetic IgG has also been developed, referred to as “bispecific diabodies”. These antibodies composed of two antigen binding Fv domains, each with a VH and a VL domains can be designed to specifically recognized a target antigen and be directed to the Fcγ receptors of cytotoxic cells in order to increase parasitic clearance (126, 128, 132). Alternatively, one of the antigen binding Fv domains can target antigen-presenting cells instead of cytotoxic cells in order to generate memory T cells and thus promote a long-lasting effect (16). If these new therapeutic approaches are effective, then it may be possible to prevent the spread of malaria altogether. However, as discussed herein, there are many important questions that remain unanswered. Taking the example of the receptor FcγRI, future studies could investigate whether the binding to monomeric antibodies hinders the recognition of the parasite’s antigens and the activation of the receptor for the destruction of immune complexes as currently presumed (133). Also, the prevalence of individuals with the FCGR1 gene lacking exon 5 and its influence in malaria resistance in endemic populations should be studied. Moreover, a different track could be pursued to understand why discrepancies have been often observed regarding the association between certain alleles and protection against malaria. For the gene FCGR2A, for example, future studies should focus on children 10 years and older, to determine the influence of the H131 allele on the production of IgG2, which increases in this age group (9, 134). It will also be important to study the hypomethylation of the gene promoter in African populations. Respectively for FCGR2C and FCGR3A, the influence of the SNP rs3933769, SNPs L66R/H, and SNP V176F should be investigated. Finally, it is important to note that different SNPs in the FCGR cluster have not yet been the target of association studies involving malaria susceptibility or resistance (**Table 2**). Indeed, further investigations to comprehensively dissect the associations between the *FCGR* cluster and malaria should be done. But, as the FCGR genes show highly homologous sequences, these studies should use next-generation sequencing and associated bioinformatic analyses.

**Table 2:** Variations of FCGR genes uninvestigated in malaria association study.

Gene	SNP	Variations	Effect on Immune System	Authors
FCGR1A	rs7531523	V39I SNP	reduces FcγRI signaling and intracellular calcium reduces immune complex binding	(71)
	rs12078005	I301M SNP	no influence on monomeric IgG reduce FcγRI signaling	(71)
	rs1050208	I338T SNP	reduces strongly FcγR signaling	(71)
FCGR2B	(-386C -120A):	-386C-120A (promoter haplotype)	affect promoter activity in both lymphoid and myeloid cell lines. Express more receptor on B lymphocytes and monocytes than -386G-120T haplotype	(103)
	rs3219018	GG386 homozygote	reduce surface expression of FcγRIIB receptors in activated B cells	(105)
FCGR2C	rs759550223	Q57X	remove expression of FCGR2C but enable expression of FcγRIIB in NK cells, that inactive FcγRIIIB	(48, 102)
	G → A (donor slicing site of intron7) G → C (acceptor splice site of intron 7)	STOP CODON	enables NK cells expressing receptor	(48)
FCGR3A	rs396991	V158F SNP	alters NK cells capacity to bind IgG1 and IgG3	(106, 107)
FCGR3B	rs403016/rs447536/rs448740/rs428888/rs2290834	NA1	greater ability to facilitate phagocytosis of opsonized particles by IgG1 and IgG3	(116)

RefSNP, accession number of SNP; Variations, SNP, allele, allotype, haplotype, genotype associated with malaria susceptibility or resistance; Effect on immune system, impact of the variation on immune response; Authors, references of the authors of the articles that mentioned the variation association.

## Author Contributions

MA wrote the first draft of the review. The other authors (AO, DO, S-PN, and WY) read and made revisions to the first draft and approved the final version for publication. All authors contributed to the article and approved the submitted version.

## Funding

This work was supported by the Developing Excellence in Leadership and Genetics Training for Malaria Elimination (DELGEME) program in sub-Saharan Africa (Grant number: PD00217ML) through the DELTAS Africa Initiative (Grant number: 107740/Z/15/Z). The DELTAS Africa Initiative is an independent funding scheme of the African Academy of Sciences (AAS)’s Alliance for Accelerating Excellence in Science in Africa (AESA) and is supported by the New Partnership for Africa’s Development Planning and Coordinating Agency (NEPAD Agency) with funding from the Wellcome Trust (Grant number: PD00217ML) and the United Kingdom government.

## Conflict of Interest

The authors declare that this research was conducted in the absence of any commercial or financial relationships that could be construed as a potential conflict of interest.

## Glossary

**Table:**

A base	adenine base
SLE	systemic lupus erythematosus
120A	alanine at position 120
386C	cysteine at position 386
ACT	Artemisinin Combination based Therapy
ASRED	allele-specific restriction enzyme digestion
ATP	adenosine triphosphate
BCR	B-cell receptor
C (1, 2 or 3)	cytoplasmic domains
CD28	cluster of differentiation 28, proteins expressed on T cells and also involved in T cell activation
CD3	cluster of differentiation 3, transmembrane protein associated with T cell receptor and zeta-chain (ζ-chain) to able T lymphocytes activation signal
CLM	clinical malaria
CM	cerebral malaria
CNV	copy number variation
cp	number of gene’ copies
D (1,2 or 3)	extracellular immunoglobulin-like domains in Fc gamma receptor
DCs	dendritic cells
DNA	deoxyribonucleic acid
EC (1or 2)	extracellular domains
*FCGR*	genes coding for Fc gamma Receptors
FcγR	receptor Fc gamma
G → A	guanine base mutated in adenine base
G base	guanine base
*H131R*	histidine substitution by arginine
HIV	Human Immunodeficiency Virus
HNA (NA)	human neutrophil antigen
HP	hyper parasitemia
*I301M*	isoleucine substitution in methionine at position 301
*I338T*	isoleucine substitution in threonine at position 338
IFN-γ	interferon gamma
IgG	immunoglobulin G
ITAM	immunoreceptor tyrosine-based activation motif
ITAMi	ITAM- mediated inhibitory signal
ITIM	immunoreceptor tyrosine-based inhibitory motif
MAPK	mitogen-activated protein kinase
MEK	MAP kinase kinases
MLPA	multiplex ligation-dependent probe amplification assay
MSP	merozoite surface protein
N82D	substitution of asparagine by aspartic acid at position 82
NGS	next generation sequencing
NK cells	Natural Killer cells
NKG2C	NK cells two C-type lectin protein
ORF	open reading frame
PCR	polymerase chain reaction
PRRs	pattern recognition receptors
PTK	protein tyrosine kinase
*Q13X*	glutamine substitution by stop codon in position 13
*Q224X*	Glutamine substitution by stop codon in position 224
*R92X*	Arginine substitution by stop codon at position 92
RefSNP	accession number of SNP
RIFIN	repetitive interspersed family proteins
RR	relative risk
S (1 or 2)	signal peptides
SH	Human neutrophil antigen-1c encoding by FCGR3B*3 allele
SH2	Src-homology 2 domain
SHIP	protein inositol phosphatase
SHP	protein tyrosine phosphatase
SM	severe malaria
SMA	severe malaria anemia
SMRT	single molecule real time
SNP	Single Nucleotide Polymorphism
SNV	Single Nucleotide Variant
Src	from “sarcoma”; non-receptor tyrosine kinase
sSA	sub-Saharan Africa
SyK	for spleen tyrosine kinase is expressed in a variety of tissues
T base	thymine base
*TLR9-1237T/C*	thymine base mutated in cytosine in position -1237 of TLR9 promoter region
*T232*	threonine at position 232 (in the transmembrane domain)
*TLR*	Toll-like receptor
*TM*	transmembrane domain
*V39I*	valine substitution in Isoleucine in position 39
*V158F*	valine substitution by phenylalanine in position 158 of the protein excluding signal peptides
*V176F*	valine substitution by phenylalanine in position 176 of the full protein
Y	tyrosine residue
βS	beta S-globin
